# B cell responses and antibody‐based therapeutic perspectives in human cancers

**DOI:** 10.1002/cnr2.2056

**Published:** 2024-03-24

**Authors:** Gunjan Mandal, Suchismita Pradhan

**Affiliations:** ^1^ Division of Cancer Biology DBT‐Institute of Life Sciences Bhubaneswar India

**Keywords:** antibody‐based immunotherapies, immunoglobulin class‐switching, tumor microenvironment, tumor‐infiltrating B lymphocytes

## Abstract

**Background:**

Immuno‐oncology has been focused on T cell‐centric approaches until the field recently started appreciating the importance of tumor‐reactive antibody production by tumor‐infiltrating plasma B cells, and the necessity of developing novel therapeutic antibodies for the treatment of different cancers.

**Recent Findings:**

B lymphocytes often infiltrate solid tumors and the extent of B cell infiltration normally correlates with stronger T cell responses while generating humoral responses against malignant progression by producing tumor antigens‐reactive antibodies that bind and coat the tumor cells and promote cytotoxic effector mechanisms, reiterating the fact that the adaptive immune system works by coordinated humoral and cellular immune responses. Isotypes, magnitude, and the effector functions of antibodies produced by the B cells within the tumor environment differ among cancer types. Interestingly, apart from binding with specific tumor antigens, antibodies produced by tumor‐infiltrating B cells could bind to some non‐specific receptors, peculiarly expressed by cancer cells. Antibody‐based immunotherapies have revolutionized the modalities of cancer treatment across the world but are still limited against hematological malignancies and a few types of solid tumor cancers with a restricted number of targets, which necessitates the expansion of the field to have newer effective targeted antibody therapeutics.

**Conclusion:**

Here, we discuss about recent understanding of the protective spontaneous antitumor humoral responses in human cancers, with an emphasis on the advancement and future perspectives of antibody‐based immunotherapies in cancer.

## 1 INTRODUCTION

It is well accepted in the field of infection immunology that the two arms of the adaptive immune system in humans, formally known as humoral and cell‐mediated immune responses, work in coordination to elicit a sustained, robust protective immune response. Surprisingly, humoral response driven by antibodies produced by tumor‐infiltrating B lymphocytes (TIL‐B) was correlated with disease progression in fast‐progressing murine models^1^, ^2^ that do not recapitulate the complex immune response network in humans. Conversely, all recent evidence correlating the extent of B cell infiltration in human solid tumors and survival of patients in multiple cancer types indicated that increased TIL‐B accumulation in the tumor microenvironment and associated humoral responses predict superior patient survival.^3^, ^4^, ^5^, ^6^, ^7^, ^8^, ^9^, ^10^, ^11^, ^12^, ^13^, ^14^, ^15^, ^16^ The effector functions of antibodies produced by the TIL‐B cells depend on the magnitude of different isotypes of antibodies produced.^17^

Studies with major solid tumor cancer types have shown that class‐switched IgG and IgA antibodies dominate the milieu of antibodies in tumor beds.^13^, ^14^, ^18^, ^19^, ^20^, ^21^ Conditions that promote one or the other type of class‐switched antibody production are still elusive. Recent reports have shown that the main antibody‐producing machinery in solid tumors is known as tertiary lymphoid structures (TLS),^22^, ^23^, ^24^ which are specialized germinal center‐like structures formed by orchestration of conglomerates of B cells and surrounding T cells.^22^, ^25^ Intratumoral TGF‐β silences Satb1, a known genomic organizer, which warrants T follicular helper cell (TFH) differentiation, and adoptive transfer of TFH cells induces intratumoral TLS formation and decreases tumor growth in vivo.^25^ One area of utmost importance is the effectiveness of these auto‐antibodies to govern disease progression. Strikingly, despite extensive spontaneous production of IgG antibodies at tumor beds, the magnitude of the response is not dramatic. In human serous ovarian cancer and endometrial cancer, although IgA is the dominant class of antibody, IgG is also extensively produced.^13^, ^14^ So far, virtually all approved therapeutic antibody preparations for clinical practice including for cancer treatment are in IgG backbone,^26^ while recently the importance of implementing dimeric IgA‐based therapeutic antibodies has been underscored.^27^, ^28^

In this review, we have summarized a comprehensive, up‐to‐date understanding of how the antibody‐producing B cells function within the tumor microenvironment, how the class‐switched antibody production is governed and its outcome, and the crosstalk of B lymphocytes with other immune cells in the tumor microenvironment. We have also discussed about major therapeutic antibodies in clinical practice and future prospects of targeting intracellular targets by specialized dimeric IgA antibodies or engineered IgG antibodies.

## 2 FUNCTION OF B CELLS AND PLASMA CELLS IN THE TUMOR MICROENVIRONMENT

Apart from a few instances where research groups have focused on IL‐10‐producing regulatory B lymphocytes in cancers^29^, ^30^ and correlated with immunosuppression,^31^, ^32^ growing converging evidence confirms that TIL‐B and their antibodyproducing plasma cell subsets predict improved outcomes of patients in multiple cancer types.^7^, ^8^, ^9^, ^13^, ^14^, ^33^ The biological function of B lymphocytes includes antigen presentation to CD4 helper T cells as antigen‐presenting cells^34^, ^35^ along with their principal function of antibody production when stimulated by cytokines, produced by activated CD4 T cells.^36^ Besides some virus‐induced cancer models,^37^ the comprehensive understanding of the antigen presentation function of B cells in solid tumor malignancies is still lacking. In epithelial ovarian cancer, increasing immunogenic advancement of the disease positively correlates with the amount of B cells in the tumor and the extent of antibody production by plasma cells.^38^ In human endometrial cancer^14^ and high‐grade serous ovarian cancer,^13^ increased infiltration of B lymphocytes has been associated with superior patient survival. Correspondingly, in other epithelial carcinomas, such as in human breast,^10^, ^21^ lung,^18^ hepatocellular,^5^ and colorectal cancers,^3^, ^4^ B cell infiltration has been associated with improved response to therapies and better survival. Equally important, B cell infiltration in non‐epithelial cancers such as sarcoma^9^ and melanoma^7^ is also a predictor of increased survival and therapy responses in patients. Helmink B. and colleagues demonstrated among melanoma patients that, individuals with higher mRNA expression for B cell‐ and antibody‐related genes such as marginal zone b and b1 cell‐specific protein, immunoglobulin lambda like polypeptide, or J‐chain, in the cancer tissues, positively correlate with response to immune checkpoint blockade therapies and survive more.^7^ Petitprez F. et al showed that B cells drive immunogenicity in soft tissue sarcoma, and they also demonstrated that B cell signatures in the tumor are associated with better responses to immunotherapies and survival outcomes in patients.^9^

The main antibody‐producing machinery in solid tumors is lymph node‐like structures formally known as TLS. Virtually in all instances, researchers have found over the years that a higher number of TIL‐B cells positively correlates with the size and density of TLS germinal centers, in different human cancer types.^39^, ^40^, ^41^ However, the types and extent of different immunoglobulin isotypes produced by TIL‐B cells are not consistent across the cancer types,^42^ and largely determine the effector functions of spontaneous humoral responses in cancer. Though others have found that plasma cells in the tumor are localized both inside and outside of the TLS,^43^, ^44^ and both in tumor islets and stroma,^45^, ^46^ Mazor and colleagues interestingly have demonstrated that tumor‐reactive IgG antibody‐producing plasma cells localize adjacent to the CD20^+^ B cell cluster in the TLS^34^. While the cornerstone for TLS formation in epithelial cancers is TFH cells,^25^, ^47^ both B lymphocytes and TFH cells get accumulated in the tumor microenvironment by a common chemokine, CXCL13^48^ because both cell types express CXCR5,^49^ the only known receptor of CXCL13.^50^, ^51^, ^52^, ^53^ The extent of antibody‐dependent cytotoxic or phagocytic killing of tumor cells and complement fixation depend on the ratio of the different antibody isotypes produced by the TLS‐orchestrated TIL‐B cells. Therapeutic interventions that enhance the formation and maturation of TLS could boost spontaneous, tumor‐destroying humoral responses.

## 3 ANTIBODY ISOTYPES, CLASS‐SWITCHING, AND THEIR FUNCTION IN SOLID TUMOR CANCERS

Antibodies, in nature, can be one of the five different classes.^54^ Naïve B cells produce IgM and IgD^55^ while antigen experiencing of B cells drives class‐switching through V‐(D)‐J recombination^56^ and production of the other three isotypes, IgG, IgA and IgE.^56^ Immunoglobulin isotype switching from constant‐μ (IgM) or constant‐δ (IgD) to downstream isotypes constant‐α (IgA) or constant‐γ (IgG) or constant‐ε (IgE) occurs by an event known as class switch recombination (CSR), which is essentially an intra‐chromosomal deletional recombination whereas the change from IgM to IgD happens through a process known as alternative splicing of the constant‐μ to constant‐δ genes.^57^, ^58^ Each isotype, except IgD, has a unique genomic region, known as the switch (S) region, upstream of each of the constant heavy regions known as the S regions. S regions (~1–12 kb long) are composed of 20‐80 bp long, G‐rich tandem repeats.^59^, ^60^ The process of CSR, occurs by end‐joining recombination, rather than by homologous recombination,^61^, ^62^ involves recombination between the S regions, and results in a change from IgM/IgD on naive B cells to one of the downstream isotypes (IgA/IgG or IgE).^59^, ^60^

Among the five antibody isotypes, IgG, IgD, and IgE only form monomers while secreted IgA and IgM form dimers and pentamers, respectively.^54^ The basic structure of all antibodies includes a pair of heavy chain and light chain.^54^ Though the antigen‐specificity of all isotypes of immunoglobulin is determined by the variable region sequences in the heavy and light chains, more precisely, by the complementarity determining regions (CDRs),^54^ their function is determined by their Fc regions which also define their isotypes.^54^ The major effector functions of antibodies are antibody‐dependent cell‐mediated cytotoxicity and phagocytosis (ADCC and ADCP) through the accumulation of cytotoxic NK cells, neutrophils, or macrophages, promoting opsonization and through activation of the complement cascade.^54^ Different antibody isotypes have different levels of abilities to drive ADCC/ADCP, opsonization or complement fixation.^54^

The two major isotypes present in human solid tumor cancers are IgG and IgA.^13^, ^14^, ^21^, ^27^, ^63^ Among the four subclasses of IgG antibodies (IgG1, IgG2, IgG3, and IgG4), IgG1 is the most efficient one for promoting ADCC/ADCP.^64^ All IgG antibodies fix complement except IgG4,^65^ while IgG2 does not function as an opsonin like other IgG subtypes.^65^ IgA is the most abundant antibody produced in humans,^66^ and it is the second most abundant in blood,^66^ while more than ninety percent of total IgA belongs to the IgA1 subclass. For polymeric IgA and IgM, the Fc chains of individual monomers are brought together by another small protein known as the J chain.^54^ A receptor expressed on the basolateral surfaces of mucosal epithelium, known as polymeric immunoglobulin receptor (PIGR) binds with the J chain of polymeric IgA/IgM^54^ and governs transcytosis of the antibody^67^ where the antibody gets complexed with a fragment of PIGR, known as a secretory component, and released from the apical surface of the mucosal epithelium.^67^ By this mechanism, polymeric IgA antibodies clear viruses from virally infected cells.^68^, ^69^ On the other hand, fetal neonatal receptor (FCRN), expressed by placental epithelium helps in transcytosis of IgG antibodies from the mother to the growing fetus.^70^, ^71^, ^72^ Recent studies suggest that PIGR (and perhaps FCRN) is expressed in several cancer tissues.^73^ While dimeric IgA binds PIGR expressed on the mucosal epithelium or cancer epithelial cells,^67^ monomeric IgA specifically binds to FcαRI (CD89)^74^ and Fcα/μR (CD351)^75^ while IgG antibodies bind to Fc gamma receptors (FcγRs).^54^ The significance of aberrant expression of some of these receptors within the tumor microenvironment is still elusive. Theoretically, some of these receptors could sequester tumor‐reactive antibodies, produced by TIL‐B cells, by re‐routing their binding with specific tumor antigens. However, binding of dimeric IgA antibodies with PIGR, which is quasi‐universally expressed by virtually all epithelial cancers, elicits transcytosis of dimeric IgA that upregulates DUSP phosphatases, which, in turn, dephosphorylates ERK1/2 and thereby dampens RAS pathway, and also sensitizes the cancer cells for MHC‐independent killing by cytotoxic T cells.^13^, ^76^ IgA‐PIGR interaction also thwarts DNA repair pathways and ER stress response proteins such as CHOP.^14^ Reportedly, CD47–SIRPα checkpoint blockade enhances tumor cell killing by neutrophils, induced by IgA.^77^ Very recently it has been demonstrated that utilizing the tumor‐penetrating abilities of dimeric IgA, intracellular mutated oncodrivers‐specific dimeric IgA antibodies could target and expel the oncoproteins out of PIGR^+^ cancer cells.^27^ The consequence of FCRN expression in cancer is yet to be clearly understood. FCRN is expressed by breast cancer cells which is retained in metastatic cells found in draining lymph nodes,^78^ and downregulation of FCRN is associated with aggressive disease progression.^79^ Similarly, in hepatocellular carcinoma^80^ and non‐small cell lung cancer,^81^ decreased FCRN expression is associated with worse outcome in patients. Interestingly, though NK cells do not express FCRN, *fcgrt* ^*−/−*^ mice show impaired NK cell development, maturation, and interferon secretion with increased melanoma metastasis into the lung.^82^, ^83^ Presumably, NK cell development and maturation need neighboring FCRN^+^ immune cells.

## 4 CROSSTALK OF B LYMPHOCYTES WITH OTHER IMMUNE CELLS WITHIN THE TUMOR MICROENVIRONMENT

**FIGURE 1:**
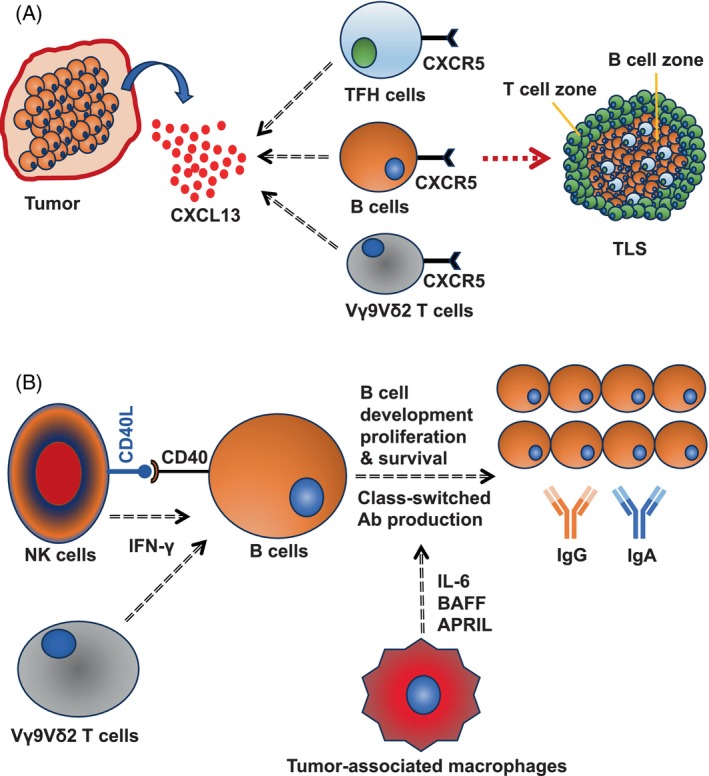
**Tumor microenvironment and B lymphocytes**. (A) CXCL13, secreted in the tumor microenvironment, attracts CXCR5^+^ B cells, CXCR5^+^ TFH cells, and CXCR5^+^ Vγ9Vδ2 γδ T cells that together promote formation of tertiary lymphoid structures. (B) NK cells promote B cell activation by engaging with CD40 on B cell surface through CD40L. NK cells and γδ T cells promote B cell class‐switching by secreting IFN‐γ. Tumor‐associated macrophages promote B cell development, proliferation, and survival by secreting B cells‐activating factor (BAFF) and a proliferation‐inducing ligand (APRIL).

B cells in the tumor microenvironment do not work alone, rather they work closely with T cells. B lymphocytes and T cells orchestrate to form specialized germinal center‐like structures, formally known as TLS. Maturation of TLS into an antibody‐producing germinal center is characterized by the presence of high endothelial venules^84^, ^85^ and requires expression of BCL6,^86^ GL7^25^ and cytokine LIGHT.^25^ In classical TLS, B cells are normally found in the center while T cells are on the periphery (Figure 1A). However, in murine ovarian cancer model, it has been demonstrated that in early developmental stages, B cells rather make the periphery surrounding the central T cell zone.^25^ The intimate crosstalk between B cells and T lymphocytes in the TLS determines the extent of humoral response within the tumor. The formation of TLS is governed mainly by the T follicular helper (TFH) cells^25^, ^47^ and chemokine CXCL13^48^ (Figure 1A). While TIL‐B cells' activity is positively correlated with effective T cell responses, B cell exhaustion often correlates with the frequency of FOXP3^+^ regulatory T cells (Tregs).^87^ Apart from interaction with T cell subsets, much is not known yet about the interactions of TIL‐B cells with other immune cells in the tumor microenvironment. Among other cells in the tumor microenvironment, NK cells could promote B cell activation and antibody class‐switching by CD40‐CD40L interaction and IFN‐γ production^88^, ^89^ (Figure 1B). A major proportion of human γδ cells, (Vγ9Vδ2 cells) express CXCR5 and therefore could co‐infiltrate the tumor microenvironment with CXCR5^+^ B cells^90^ (Figure 1A). Similar to NK cells, γδ cells also promote class‐switched antibody production^90^, ^91^ (Figure 1B). Nonetheless, tumor‐associated macrophages (TAMs) secrete B cells‐activating factor (BAFF) and a proliferation‐inducing ligand (APRIL) and govern B cell proliferation,^92^ and also support B cell development by secreting IL‐6^93^ (Figure 1B). Chen Z et al found that TAMs drive IgG‐producing plasma cells in hepatocellular carcinoma.^63^ In a very intriguing study, Shaul M et al have demonstrated that the differentiation of tumor‐infiltrating CD45^+^B220^+^CD138^−^ B cells into IgG‐producing B220^+^CD138^+^ plasma cells requires physical contact with tumor‐associated neutrophils, which they found to be independent of the involvement of tumor‐infiltrating T cells.^94^ Notably, they have also found that tumor‐associated neutrophils drive B cell recruitment to the tumor microenvironment by secreting TNF‐α.^94^ In a mouse model of lung cancer, Wang Y et al demonstrated that with an increasing number of myeloid‐derived suppressor cells (MDSCs)‐infiltration into the tumor mass they observed impaired B cell functions, indicated by decreased serum IgG levels.^95^ MDSCs^96^ in the tumor microenvironment force B cell differentiation into IL‐10, IL‐35, and TGF‐β‐producing^97^ immunosuppressive regulatory B cells (Bregs),^98^, ^99^ however, Bregs do not have any unique identification marker like FOXP3 for Tregs, and therefore the classification of Bregs among total B cells is elusive.

## 5 THERAPEUTIC ANTIBODIES IN THE CLINICS AND FUTURE PERSPECTIVES

Despite the huge early success of two monoclonal antibody‐based immunotherapies for cancer, rituximab^100^, ^101^ and trastuzumab^102^, ^103^ against CD20‐positive B cell lymphoma and Her2^positive^ estrogen receptor (ER)/progesterone receptor (PR)^negative^ breast cancer, respectively, the field of immunotherapy research gradually became more focused on cellular therapies such as adoptive TIL therapy,^104^, ^105^ CAR T cells therapy,^106^, ^107^ CAR NK therapy^108^, ^109^ etc. after their success in solid tumors. The importance and effectiveness of these cellular therapies are unquestionable, however, the cost of such treatments is very expensive in their current states^110^ while antibody‐based therapies are significantly cost‐effective and therefore affordable to a larger proportion of eligible patients. Primarily the effector functions of immunotherapeutic antibodies against cancer lie in their ability to induce ADCC/ADCP or complement fixation. Rituximab, a monoclonal IgG1 antibody, binds with CD20 on malignant B cells and induces NK cells‐ and macrophage‐mediated killing or complement‐mediated lysis of the cancer cells.^111^ Similarly, Daratumumab, a monoclonal IgG1 antibody, binds with CD38 on myeloma cells and is extensively used for treating multiple myeloma patients.^112^ Resistance to rituximab in relapsed refractory lymphoma patients is common^113^ and therefore alternative approaches have been introduced later in the clinical practice, for example targeting CD79b by Polatuzumab^114^ or using bi‐specific antibodies, such as Glofitamab which is a CD20‐directed, CD3 T cells engager.^115^, ^116^ Trastuzumab, which is also an IgG1 monoclonal antibody, works in a similar fashion to Rituximab or Daratumumab by binding with surface protein Her2 on breast cancer cells and is a life‐saving drug for ER/PR^negative^ Her2^positive^ breast cancer patients.^117^ Apart from killing target cancer cells, rescuing antitumor cytotoxic functions of TILs resulted in significant increase in antitumor immunities and corresponding reduction in cancer progression by targeting CTLA‐4^118^, ^119^ and PD‐1^120^, ^121^ by monoclonal antibodies were demonstrated in parallel by James P. Allison and colleagues; and Tasuku Honjo and associates, respectively, popularly known as immune checkpoint blockade (ICB) therapies, which produced the Joint‐Nobel prize in physiology or medicine in the year 2018.^122^ The anti‐PD‐1 antibodies (Nivolumab or Pembrolizumab) are made in IgG4 backbone,^123^, ^124^ to annihilate the elimination of antibody‐bound PD‐1 positive T cells by ADCC/ADCP or complement, though the anti‐CTLA‐4 antibody, Ipilimumab, was developed in human IgG1 backbone.^125^

**FIGURE 2:**
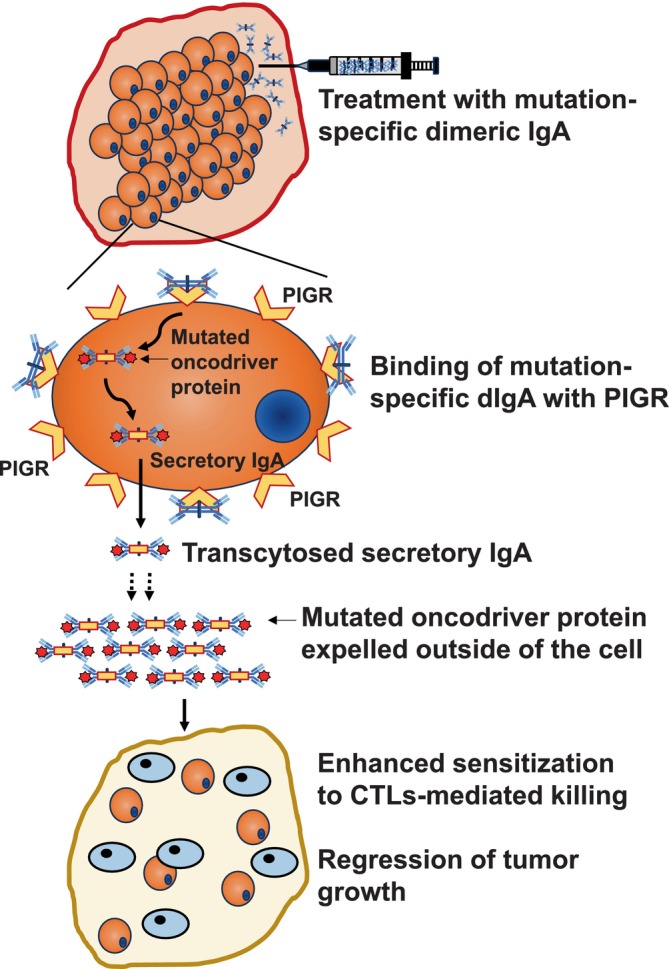
**Intracellular targeting of mutated oncoproteins by dimeric IgA antibodies**. Mutation‐specific dimeric IgA antibodies can bind with PIGR expressed on the epithelial cancer cells of major cancer types and get internalized with a fragment of PIGR known as the secretory component. Internalized dimeric IgA antibodies encounter target antigens in the cytoplasm and could haul them outside of the cells through transcytosis whereby the mutated oncodrivers could be expelled into the extracellular space by secretory IgA. Treatment of PIGR^+^ tumor cells sensitizes them for CD8^+^ cytotoxic T cell‐mediated killing.

The field started considering antibody‐mediated immunotherapies more seriously for solid tumor cancer treatments after the success of checkpoint inhibitor monoclonal antibodies in the clinics against PD‐1, CTLA‐4, and PD‐L1 (ligand of PD‐1).^126^, ^127^, ^128^, ^129^ Approaches for targeting intracellular oncoproteins and mutated oncodrivers have been restricted to the development of novel small molecule inhibitors.^130^ Some of them are like different EGFR inhibitors.^131^ recently FDA‐approved Sotorasib^132^ targeting G12C‐mutated KRAS or MRTX1133^133^ targeting KRAS^G12D^ which is awaiting FDA‐approval. Unfortunately, small‐molecule inhibitors have a smaller half‐life than antibodies^134^ and the development of resistance is very common.^135^, ^136^ Dimeric IgA antibodies, which bind naturally expressed PIGR^137^ on the basolateral surface of mucosal epithelium, through their J‐chain,^54^ and undergo transcytosis by taking a portion of PIGR (known as secretory component), and released by the apical surface,^67^ and through this mechanism they clear viruses from the gut.^68^, ^69^ Notably, PIGR is heavily expressed in a majority of the epithelial cancer types and it has been exhibited that dimeric IgA can also undergo PIGR‐driven transcytosis through cancer epithelial cells,^13^, ^14^ which instigated their use for targeting intracellular oncoproteins. Recent findings demonstrated that specific dimeric IgA antibodies, but not the same antibodies in IgG backbone, against G12D mutation of KRAS and R132H mutation of IDH1, could target KRAS^G12D^ and IDH1^R132H^, respectively, inside the cancer cells harboring these mutations and significantly abrogate tumor progression in vivo^27^, ^138^ (Figure 2). This opens a novel opportunity to develop dimeric IgA‐based (or perhaps engineered IgG with the ability to bind and internalize with PIGR) therapeutic antibodies against virtually any intracellular oncoproteins. In future, strategies such as developing antibodies permeable through the cell membrane and reachable to the subcellular locations like the nucleus (for example against mutant p53) could shift the paradigm of antibody‐bases immunotherapies against cancer and other diseases.

## 6 CONCLUDING REMARKS

Though the role of T cells in antitumor immunity is indisputable, it is time to appreciate the importance of coordinated humoral and cellular immune response in human malignancies and look for strategies that induce sustained, robust antitumor immune responses by driving simultaneously both the arms of immune system. Apart from the suitability of targeting intracellular targets by dimeric IgA antibodies, their suitability to synergize with existing immunotherapies and chemotherapies should also be evaluated. Previously it has been shown that non‐antigen specific dimeric IgA could enhance T cell‐mediated killing of PIGR‐expressing cancer cells in an MHC‐I independent manner, therefore, there is a high chance that therapeutic use of dimeric IgA antibodies could also promote cytotoxic killing by CD8 T cells, in addition to ADCC/ADCP. The development of penetrable IgG antibodies is also important, pertinent to the fact that IgG antibodies have a much higher half‐life, and therefore industrial production of monomeric IgG is cost‐effective than dimeric IgA antibodies.

## AUTHOR CONTRIBUTIONS

**Gunjan Mandal:** Conceptualization; funding acquisition; writing – original draft; supervision; visualization; writing – review and editing; methodology. **Suchismita Pradhan:** Writing – original draft; writing – review and editing.

## CONFLICT OF INTEREST STATEMENT

The authors do not have any conflict of interest.

## Data Availability

Data sharing is not applicable to this article as no new data were created or analyzed in this study.
